# Pressurized Intraperitoneal Aerosol Chemotherapy (PIPAC) in the Treatment of Gastric Cancer: Feasibility, Efficacy and Safety—A Systematic Review and Meta-Analysis

**DOI:** 10.3390/jcm13113320

**Published:** 2024-06-04

**Authors:** Francisca Ramalho-Vasconcelos, Raquel Gomes, Raquel Bouça-Machado, Marisa Aral, Jorge Nogueiro, Tiago Bouça-Machado, Bernardo Sousa-Pinto, Hugo Santos-Sousa

**Affiliations:** 1 Faculty of Medicine, University of Porto, 4200-319 Porto, Portugal; 2 Instituto de Medicina Molecular João Lobo Antunes—Edifício Egas Moniz, Avenida Professor Egas Moniz, 1649-028 Lisboa, Portugal; 3 São João Local Health Unit, Surgery Department, 4200-319 Porto, Portugal; 4 MEDCIDS—Department of Community Medicine, Information and Health Decision Sciences, Faculty of Medicine, University of Porto, 4200-319 Porto, Portugal; 5 CINTESIS—Centre for Health Technologies and Services Research, University of Porto, 4200-319 Porto, Portugal; 6 São João Local Health Unit, Obesity Integrated Responsibility Unit (CRI-O), 4200-319 Porto, Portugal

**Keywords:** gastric cancer, gastric adenocarcinoma, stomach cancer, gastric neoplasm, peritoneal carcinomatosis, peritoneal metastasis, Pressurized Intraperitoneal Aerosol Chemotherapy, PIPAC, systematic review, meta-analysis

## Abstract

**Background:** Pressurized Intraperitoneal Aerosol Chemotherapy (PIPAC) is an emerging technique for delivering chemotherapy directly to the peritoneum via a pressurized aerosol. Its growing attention stems from its effectiveness in treating peritoneal carcinomatosis (PC) originating from various primary tumors, with gastric cancer (GC) being among the most prevalent. This study aimed to systematically investigate PIPAC’s therapeutic role in gastric cancer peritoneal metastasis (GCPM). **Methods:** The systematic review and meta-analysis followed the PRISMA 2020 guidelines, searching Pubmed, Web of Science, and SCOPUS databases. The meta-analysis of relative risks and mean differences compared patients undergoing one or two PIPAC sessions with those completing three or more, assessing various outcomes. **Results:** Eighteen studies underwent qualitative analysis, and four underwent quantitative analysis. Patients with three or more PIPAC procedures had shorter hospital stays (MD = −1.2; 95%CI (−1.9; −0.5); *p* < 0.001), higher rates of histopathological response (RR = 1.77, 95%CI 1.08; 2.90; *p* = 0.023), and significantly improved overall survival (MD = 6.0; 95%CI 4.2; 7.8; *p* < 0.001). Other outcomes showed no significant differences. **Conclusions:** PIPAC demonstrated efficacy in carefully selected patients, enhancing histopathologic response rates and overall survival without prolonging hospital stays. This study underscores the necessity for randomized controlled trials and precise selection criteria to refine PIPAC’s implementation in clinical practice.

## 1. Introduction

Gastric cancer (GC) incidence and mortality rates have exhibited a gradual decline over recent decades, particularly in developed countries. Despite this trend, GC remains a significant contributor and an important cause of cancer morbidity and mortality, characterized by an ongoing unfavorable prognosis [1,2]. Notably, it was estimated to rank as the fifth-most prevalent cancer globally in 2020, contributing to 8.0% of all cancer-related deaths [1,3]. The diagnosis rate has been approximated at 6.9 per 100,000 new cases annually. As of 2023, an estimated 26,500 new cases of gastric cancer are projected, with approximately 11,130 resultant fatalities, accounting for 1.8% of all cancer-related deaths [4].

In specific regions, particularly in Eastern Asia and Eastern Europe, gastric cancer ranks as the third-most frequently diagnosed cancer. However, its prognosis continues to be bleak due to usual late-stage diagnoses [1,3,5]. Peritoneal metastases are notably prevalent, occurring in 15 to 30.0% of patients at initial diagnosis. Additionally, among patients treated with curative intent (via gastrectomy and systemic chemotherapy), 40 to 60.0% experience peritoneal metastasis as the sole site of recurrence [3,6,7].

Standard treatment for metastatic gastric cancer predominantly involves palliative systemic chemotherapy, yielding a limited prognosis with a median overall survival of 6 months [3,8,9]. Despite advances in treatment modalities aimed at enhancing survival rates, progress has been incremental, achieving a survival range of 8–14 months in select cases [8,10].

Gastric cancer with peritoneal metastasis (GCPM) presents considerable therapeutic challenges, due to the extensive surface area of the peritoneal cavity and the poor vascularization of the peritoneum. These factors severely restrict the efficacy of systemic chemotherapy in eradicating peritoneal metastases [11,12]. Consequently, novel strategies employing intraperitoneal drug delivery have emerged as potential innovative approaches to address these limitations, such as normothermic intraperitoneal chemotherapy, hyperthermic intraperitoneal chemotherapy (HIPEC), normally combined with cytoreductive surgery (CRS) and, more recently, pressurized intraperitoneal aerosolized chemotherapy (PIPAC) [13,14,15,16].

PIPAC was first described in 2011 as a novel way to deliver chemotherapy directly into the peritoneum as a pressurized aerosol [17,18]. In fact, it represents a minimally invasive therapeutic approach, designed to enhance intraperitoneal drug delivery by taking advantage of fundamental physical principles. This methodological innovation aims to optimize the uniformity of drug distribution within the peritoneal cavity, by employing an aerosolized form rather than a liquid solution. Additionally, PIPAC utilizes augmented intraperitoneal hydrostatic pressure to counteract heightened intertumoral interstitial fluid pressure, thereby enhancing drug penetration. Further strategies involve the restriction of blood outflow during drug administration and the modulation of environmental factors within the peritoneal cavity (such as temperature, pH, and electrostatic charge) to optimize tissue-specific targeting effects.

The technique of PIPAC involves the laparoscopic insertion of two balloon trocars in an operating room with laminar airflow conditions. Initially, a normothermic pneumoperitoneum is established, and maintained at a pressure of 12 mmHg. Following this, a cytotoxic solution, typically constituting 10–20% of a standard systemic dosage, is nebulized into the abdominal cavity using a micropump, where it remains for a duration of 30 min. Subsequently, the pneumoperitoneum is removed from the peritoneal cavity through a closed suction system [11,12].

Moreover, PIPAC offers the advantage of facilitating repeated applications of chemotherapy while enabling an objective evaluation of tumor response through comparative biopsies conducted between chemotherapy cycles [11].

However, despite the promising findings presented by several ongoing phase I and phase II clinical trials, the application of PIPAC in managing GCPM remains a topic of debate [8]. Furthermore, there is a scarcity of current studies investigating this approach, particularly in the context of GCPM patients.

Therefore, this systematic review aims to evaluate the influence of PIPAC on the management of patients diagnosed with GCPM. Specifically, the objective consisted in assessing the feasibility, efficacy, and safety of this procedure.

## 2. Materials and Methods

This systematic review and meta-analysis was conducted according to the Preferred Reporting Items for Systematic Reviews and Meta-Analyses (The PRISMA 2020 Statement) [19] and to the Cochrane Handbook for Systematic Reviews [20]. This systematic review was also registered in the International Prospective Register of Systematic Reviews PROSPERO [21] as CRD42023428017 [22].

### 2.1. Eligibility Criteria

In this review, papers were eligible if they assessed adult patients (patients over 18 years old) diagnosed with gastric cancer with peritoneal carcinomatosis, who underwent PIPAC treatment (with or without concomitant systemic chemotherapy). Prospective and retrospective cohorts, case–controls, and single-arm clinical trials were included. Case series, case reports, and survey-based studies were excluded. Studies that performed in vitro or in animal research, studies on environmental safety, meeting abstracts, and comments and correspondence letters were also excluded. Studies written in a non-English language were considered non-eligible after attempting and failing to contact the authors of the studies in question requesting an English written version.

Studies were also excluded if they only assessed the role of PIPAC treatment for primary tumors other than gastric cancer with PM. If studies included the assessment of other primary tumors with PM, but they also included the assessment of the treatment of GC with PM, data concerning exclusively the demographics, the treatment, and outcomes of patients with GCPM was retrieved and included in this systematic review. Studies focusing on alternative methods of intraperitoneal chemotherapy such as HIPEC and other methods of intraperitoneal delivery were also excluded.

### 2.2. Search Strategy, Information Sources and Selection Process

The search was first conducted in March 2023 by two independent investigators (FRV and RG) using three different electronic databases: PubMed, Web of Science and SCOPUS. The search was performed using the queries shown in Supplementary Table S1. In addition, citation searching was also performed, concerning the reference lists of the primary articles. All articles which were generated from the electronic search were imported into Rayyan© (Qatar Foundation, Doha, Qatar), an online tool used in the management of systematic reviews. After removal of duplicates, the selection process was conducted in two phases. In the first phase, the researchers screened all articles, and selected the studies based on their titles and abstracts. In the second phase, articles were selected based on the full text reading, performed by FRV and RG. Any disagreements were solved by a third investigator (HSS).

The search was limited to studies published between 1 January 2011 and 8 March 2023, since the concept was first introduced in 2011 by Solass et al. [17,18].

### 2.3. Aims

The primary objective of the present review was to evaluate the effectiveness, feasibility, and safety of PIPAC as a therapeutic intervention for GC patients with PM.

Assessing the efficacy of PIPAC included evaluation of the histopathological response, determined by analyzing the histologic grading score, the Peritoneal Regression Grading Score (PRGS) [23], after each PIPAC. The variable was calculated as the number of patients who developed a PRGS of 1 or 2, within the total patient cohort considered for that specific outcome in each respective study. Additionally, estimates were made regarding macroscopic and clinical response, based on the Peritoneal Cancer Index (PCI), with the variable defined as the number of patients exhibiting a PCI of 12 or higher [24,25], and changes in ascites volume.

Feasibility was evaluated by examining both the non-access rate to the abdominal cavity and the proportion of patients who successfully completed three or more cycles of PIPAC. Quantitative analysis regarding the length of hospitalization and operative time were also considered in feasibility.

The safety assessment focused on the occurrence of adverse events, as classified by the Common Terminology Criteria for Adverse Events (CTCAE), and the occurrence of surgical complications according to the Clavien–Dindo classification. A quantitative analysis was performed regarding the overall occurrence of adverse events using CTCAE.

Finally, a survival analysis was conducted, regarding the overall survival from the first PIPAC treatment. Median overall survival concerning GC patients was considered for each study, when available. Estimated survival (%) at 6, 12, 18 months and 24 months were also considered. Finally, a quantitative analysis regarding mean overall survival for each subgroup was also attempted.

### 2.4. Data Collection Process

The criteria regarding data extraction were established at the beginning of the review process. Data were also retrieved by two independent reviewers (FRV and RG). The established criteria were approved by all three reviewers beforehand. Afterwards, the two independent reviewers (FRV and RG) extracted data into a predesigned data extraction form that was developed according to the agreed criteria and the Cochrane Handbook [20].

From each study, information such as authors and year of publication, country, study design, data collection period, type of PIPAC chemotherapy, number of PIPAC sessions, percentage of non-access to the abdominal cavity, percentage of patients who completed 3 or more PIPAC cycles, sample size, sex percentage, mean age and PCI at the first procedure, percentage of patients who present with ascites at the first PIPAC, and mean volume, mean operative time, length of hospital stay and follow-up, primary tumor origin, and PIPAC treatment regimen was extracted.

### 2.5. Quality and Risk of Bias Assessment

The methodological quality regarding the included studies was assessed by two independent reviewers (FRV and RG) using the National Institutes of Health Quality Assessment Criteria for observational and case–control studies and before–after (pre–post) studies with no control group [26]. The Quality Assessment Criteria for observational studies consists of a series of 14 “Yes” or “No” questions. Both the Quality Assessment Criteria for case–control studies and before–after (pre–post) studies include a series of 12 different “Yes” or “No” questions. Application of all three Quality Assessment Criteria result in a rating of “good”, “fair”, or “poor” for each study. Finally, when the matter in question is not applicable nor referred, the answer applied was not applicable/not referred.

### 2.6. Missing Data

In instances where information was not available, the corresponding authors of the referenced studies were contacted to obtain the necessary details.

### 2.7. Quantitative Synthesis of Results

Moreover, a random-effects meta-analysis was conducted, utilizing the restricted maximum likelihood approach to assess mean differences (MD) for continuous outcomes, including operative time, length of hospitalization, variation in ascites volume, and overall survival. Additionally, relative risks (RR) were analyzed for dichotomous variables such as PRGS (1 or 2), occurrence of adverse events, and PCI (12 or higher).

Heterogeneity was assessed using Cochran’s Q statistic *p*-value and the I^2^ statistic. High heterogeneity was defined as I^2^ > 50% or Cochran’s Q test *p*-value < 0.10.

A *p*-value below 0.05 was considered to indicate statistical significance. Furthermore, the meta-analysis was conducted using the meta package in the R software Version 4.3.

## 3. Results

### 3.1. Study Selection

**Figure 1:**
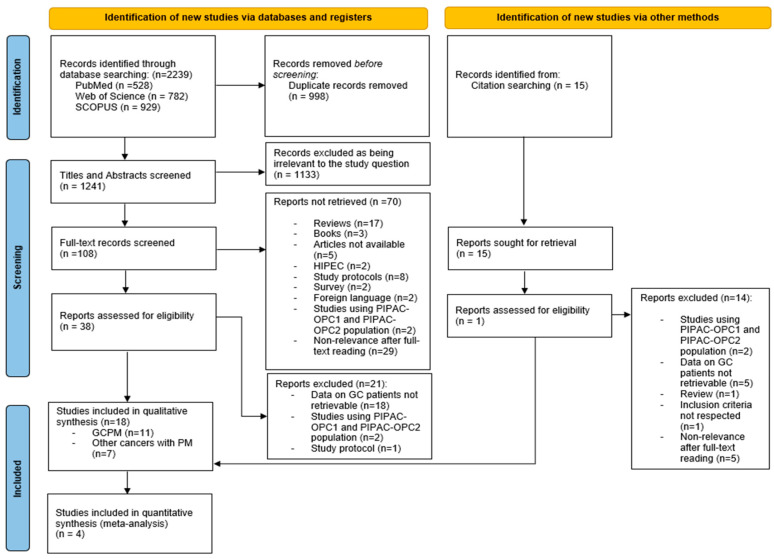
Flow diagram of study selection.

Following the search of the three databases previously referred to, a total of 2239 studies were found, of which 998 were duplicates. After the removal of duplicates, a total of 1241 articles were screened based on their title and abstract. A total of 1133 studies were excluded since they were irrelevant to the study question. Afterwards, a total of 108 were screened based on their full text, and 38 were assessed for eligibility, of which 17 were included. Citation searching resulted in one additional article. Therefore, a total of 18 articles were included in the qualitative synthesis, 4 of which were further included in the quantitative synthesis (meta-analysis). Eleven studies concerned a cohort of GCPM patients and seven studies included a cohort of patients with PM of several tumor origins besides gastric (colorectal, pancreatic, ovarian, appendiceal, biliary, esophageal, small bowel, uterine, mesothelioma, etc.). In relation to these seven included studies, only data pertaining to patients with GCPM were considered for integration into the present review. During the database search, several additional articles were identified that addressed PM from various primary tumors, including GC. However, these studies did not provide data exclusively concerning GCPM patients. Consequently, these studies were excluded and categorized as “Data on GC patients not retrievable”, as depicted in Figure 1. Furthermore, the population of the studies PIPAC-OPC1 and PIPAC-OPC2 resulted in seven distinct publications [27,28,29,30,31,32,33]. Of those seven publications, only Ellebæk et al. was included in the present review [33]. The study selection process is summarized in Figure 1 [19].

### 3.2. Study Characteristics

The characteristics of all the included studies in this systematic review are represented in Table 1. Regarding the 18 studies included, 10 were retrospective cohorts [34,35,36,37,38,39,40,41,42,43], 4 were prospective cohorts [33,44,45,46], 1 was a retrospective case–control study [47], and 3 were single-arm clinical trials (intervention studies) [48,49,50].

These studies were published between 2015 and 2023 and were conducted in Germany, Denmark, Italy, France, India, Russia, and Lithuania. Two studies were multicentric (conducted in three and five institutions, respectively) [41,46].

A total of 440 patients were studied with sample sizes ranging from 1—information concerning a single GCPM patient was extracted from a study addressing several primary tumors which developed PM—to 144. Almost all studies used cisplatin (7.5 mg/m^2^ of body surface in 150 mL NaCl 0.9%) plus doxorubicin (1.5 mg/m^2^ in 50 mL NaCl 0.9%) as PIPAC chemotherapy. One study opted to administer a higher dosage of cisplatin, plus the previously mentioned dose of doxorubicin (cisplatin (15 mg/m^2^) plus doxorubicin (1.5 mg/m^2^)) [43]. Another study combined the referred chemotherapy with electrostatic precipitation in a subset of patients. In fact, during this procedure—called ePIPAC—after the intraperitoneal delivery of the drug, the Ultravision generator (Ultravision, Alesi Surgical Ltd., Cardiff, UK) is turned on, and electrostatic precipitation is performed for at least one minute [33].

The number of PIPAC sessions conducted in the included studies ranged from 2 to 296, and the mean PCI at the first PIPAC varied between 5.0 and 23.5 ± 8.7. The shortest mean follow-up time was 8.4 ± 4.8 months and the longest 16.1 ± 12.0 months.

Eleven of the included studies were conducted on patients with gastric cancer being the primary tumor, while the remaining seven studies included a population of several neoplasms with peritoneal carcinomatosis. Regarding PIPAC regimen, most studies focused solely on PIPAC administration, not specifying if there was concomitant systemic chemotherapy administered in that population. Nonetheless, some studies approach PIPAC as a concomitant therapy: one study focused on the results of PIPAC administration alternately with systemic chemotherapy versus systemic chemotherapy alone [47]. A phase II clinical trial developed a study protocol combining PIPAC (low-dose cisplatin and doxorubicin) with systemic combination chemotherapy (oxaliplatin and capecitabin: XELOX protocol) [48]. Another study aimed to determine the safety of PIPAC surgery following VEGFR2 antagonist, ramucirumab-containing chemotherapy, as a second line treatment for GCPM [37]. Additionally, from the 18 included studies, after being submitted to PIPAC chemotherapy, a total of 18 patients became eligible to receive CRS-HIPEC.

Table 1 represents the characteristics mentioned of the included studies.

### 3.3. Quality Assessment and Risk of Bias of Individual Studies

The quality assessment and risk of bias is shown in Supplementary Tables S2–S4. All studies fail to justify sample size established or the assessors being blinded. Only four studies succeeded in measuring and adjusting potential confounding variables. Six studies did not examine different levels of exposure as related to the outcome, since in these studies PIPAC exposure was only assessed once. For the remaining criteria, most of the studies scored a low risk of bias. Consequently, all studies were considered “Good” except one, which scored “Fair”.

### 3.4. Feasibility

With regard to the non-access rate to the abdominal cavity, when reported, the majority of studies indicate a non-access rate of 0.0%, with the highest reported rate being 13.9%. The percentage of patients who completed three or more procedures exhibits substantial variability across different studies. Regarding the mean operative time, variations were observed, ranging from 62.3 ± 14.1 min to 113.8 ± 38.1 min, as reported by Horvath et al. in 2022. Moreover, as for Ellebæk et al., and concerning the ePIPAC procedure alone, the mean operative time was 71.5 ± 13.9 min. Lastly, concerning the mean hospital stay for patients, the duration spanned from 2.0 to 16.0 ± 12.4 days. Table 2 provides a detailed presentation of the previously stated information.

### 3.5. Safety

Out of the 18 studies analyzed in this systematic review, 12 studies provide details on adverse events associated with the procedure, utilizing either the CTCAE or Clavien–Dindo classification. The occurrence of CTCAE grade 3 or 4 adverse events ranged from 0.0% to 29.2%, with a notable proportion of studies reporting zero events. Regarding CTCAE grade 5 events, the vast majority of studies reported no occurrences, with the highest documented incidence being 8.3% in the study by Nadiradze et al. Finally, considering the Clavien–Dindo classification, events of grade 3a, 3b, or 4 varied from 0.0% to 11.8%. A detailed summary of this information is presented in Table 3.

### 3.6. Survival Analysis

As for the overall survival of patients submitted to PIPAC procedures, a comprehensive analysis of 12 studies revealed a median overall survival ranging from 6.0 months, as reported in two separate studies (6.0 (1.4–21.2) [39] and 6.0 (2.9–15.5) [42]), to 15.4 months. The survival rates at 6 months were reported by 13 studies, ranging from 55.6% to 100.0%. At the end of the first year, the survival rate exhibited a range from 0.0% to 73.5%. The overall survival at 18 months displayed considerable variability, spanning from 0.0% to 55.7%. Although few studies reported survival rates at 2 years, the highest reported rate was 39.4. Further details are provided in Table 4.

### 3.7. Comparison between 1 or 2 PIPAC and 3 or More PIPAC

#### 3.7.1. Feasibility

A total of 188 patients were analyzed in two distinct studies, regarding the operative time of the PIPAC procedure. No significant differences between the number of procedures (one or two and three or more) executed were observed (MD = 0.3, 95%CI (−9.4; 10.0); *p* = 0.0955) and high heterogeneity was detected (I^2^ = 64%; *p* = 0.10).

**Figure 2:**
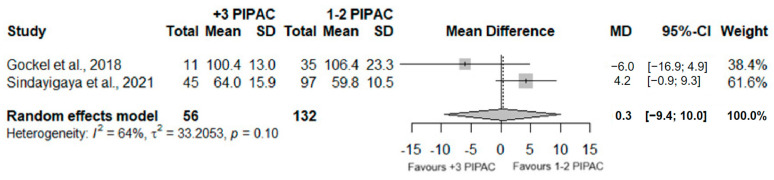
Forest plot of the comparison between 1 or 2 PIPAC and 3 or more PIPAC, regarding operative time [38,44].

**Figure 3:**
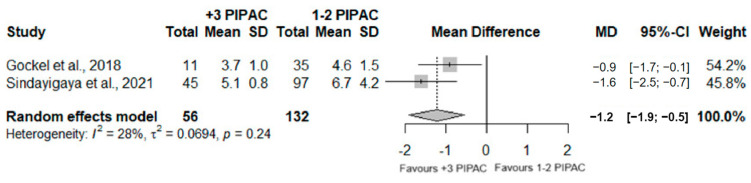
Forest plot of the comparison between 1 or 2 PIPAC and 3 or more PIPAC, regarding hospital stay duration [38,44].

Two studies examined the length of hospitalization of patients who underwent PIPAC surgery, involving a total of 188 analyzed patients. A significant difference was observed (MD = −1.2; 95%CI (−1.9; −0.5); *p* < 0.001), with the group who underwent three or more PIPAC having a lower period of hospitalization, when comparing with patients who underwent one or two PIPAC. Furthermore, moderated heterogeneity was observed (I^2^ = 28%; *p* = 0.24). Figure 2 and Figure 3 illustrate the information previously detailed.

#### 3.7.2. Safety

**Figure 4:**
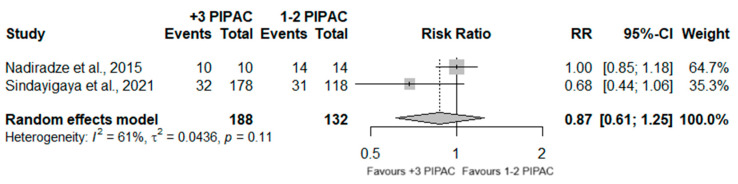
Forest plot of the comparison between 1 or 2 PIPAC and 3 or more PIPAC, regarding the occurrence of overall adverse events (CTCAE criteria) [36,38].

Two studies reported that the overall adverse events, according to CTCAE criteria, occurred in a total of 320 patients after receiving PIPAC surgery. No significant differences were observed between the two groups (RR = 0.87; 95%CI (0.61; 1.25); *p* = 0.460), although high heterogeneity was observed (I^2^ = 61%; *p* = 0.11) (Figure 4).

#### 3.7.3. Efficacy

**Figure 5:**
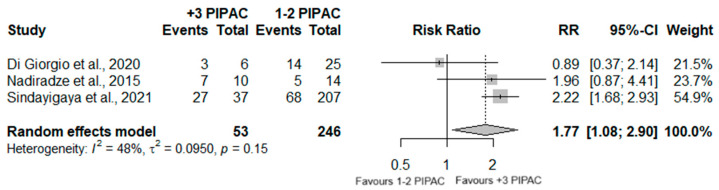
Forest plot of the comparison between 1 or 2 PIPAC and 3 or more PIPAC, regarding PRGS =1 or 2 [35,36,38].

A total of 299 patients were analyzed regarding the PRGS score, in three distinct studies. Significant differences were observed between the two groups (RR = 1.77 95%CI (1.08; 2.90); *p* = 0.023), with the group who underwent three or more PIPAC procedures having a significantly higher frequency of a complete/major histologic regression. Nonetheless, moderated heterogeneity was observed (I^2^ = 48%; *p* = 0.15) (Figure 5).

**Figure 6:**
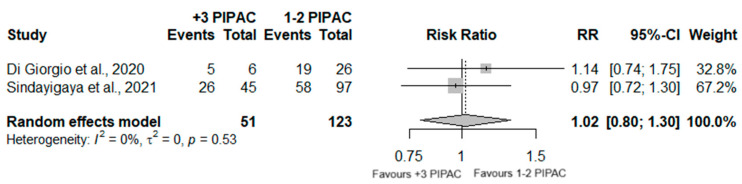
Forest plot of the comparison between 1 or 2 PIPAC and 3 or more PIPAC, regarding PCI = 12 or higher [35,38].

A total of 174 patients were analyzed in two different studies, considering the PCI cutoff of 12 or higher, between the two groups. However, no significant differences between the two groups were observed (RR = 1.02; 95%CI (0.80; 1.30); *p* = 0.873) and no heterogeneity was detected (I^2^ = 0%; *p* = 0.53) (Figure 6).

**Figure 7:**
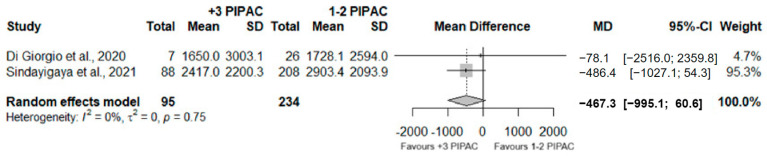
Forest plot of the comparison between 1 or 2 PIPAC and 3 or more PIPAC, regarding ascites volume variation [35,38].

Regarding the ascites volume variation between the two groups, a total of 329 were included. No significant differences between the two groups were observed (MD = −467.3; 95%CI (−995.1; 60.6); *p* = 0.083) and no heterogeneity was observed (I^2^ = 0%; *p* = 0.75) (Figure 7).

#### 3.7.4. Survival Analysis

**Figure 8:**
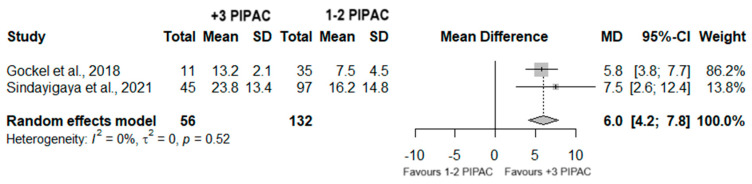
Forest plot of the comparison between 1 or 2 PIPAC and 3 or more PIPAC, regarding the mean OS [38,44].

A total of 188 patients were included from two different studies, when evaluating the mean overall survival. In fact, a significant difference was observed (MD = 6.0; 95%CI (4.2; 7.8); *p* < 0.001) with the group who underwent three or more PIPAC procedures exhibiting a higher mean overall survival. Furthermore, no heterogeneity was observed (I^2^ = 0%; *p* = 0.52) (Figure 8).

## 4. Discussion

Individuals exhibiting peritoneal metastasis (PM) demonstrate decreased survival rates compared to those with parenchymatous metastasis, such as individuals experiencing liver dissemination [51]. The unfavorable prognosis in PM cases is attributed to multiple factors, including resistance to cytotoxic drugs, limited tolerance to chemotherapy, accelerated decline in patient performance, and intestinal dysfunction linked to tumor invasion of the bowel [11,51].

Especially in the context of advanced GC, where the standard of care involves doublet or triplet systemic chemotherapy, the effectiveness of these regimens in securing prolonged survival for patients with peritoneal metastasis remains “modest at best” [11,52]. In fact, it is generally acknowledged that the current treatment options for PM are limited and that outcomes are poor [53].

PIPAC, a palliative surgical approach designed for the pressurized delivery of chemotherapy agents (cisplatin, doxorubicin, oxaliplatin) into the peritoneum, has recently been incorporated into the repertoire of oncological interventions for addressing peritoneal metastasis in individuals ineligible for cytoreductive surgery or HIPEC [54,55]. The initial documentation of successful PIPAC application in three patients with peritoneal metastasis dates back to 2014 [18], and subsequently, a limited number of publications have outlined the efficacy and safety of PIPAC in managing peritoneal metastasis across diverse cancer types, with gastric cancer being one of the most frequently reported [56].

Regarding the topic of PIPAC in the treatment of PM, a total of eleven systematic reviews [53,56,57,58,59,60,61,62,63,64,65] and six narrative reviews [55,66,67,68,69,70] have been published. The most recent systematic review, by Di Giorgio et al., was published in 2023 [53]. The mentioned study, which concerned a cohort of several primary tumor origins who developed peritoneal carcinomatosis, concluded that PIPAC exhibits promise as a treatment for specific peritoneal metastasis patients, demonstrating acceptable toxicity and potential survival benefits. However, inconsistencies in data reporting across studies raise concerns about result reliability. Subset analyses reveal significant tumor regression, implying the cytotoxic effectiveness of PIPAC. The study underscores the urgent need for standardized patient selection, reporting criteria, and clinical endpoints to ensure reliable cross-study comparisons and robust results.

Furthermore, with an exclusive emphasis on the management of PM originating from gastric cancer, the available literature comprises only two systematic reviews [56,59] and one narrative review [55]. The most recent systematic review on this subject was conducted by Case et al. in 2022 [59]. Their findings indicate that PIPAC holds potential as a therapeutic intervention for specific patients with peritoneal carcinomatosis arising from gastric cancer, showing promising survival benefits, acceptable toxicity levels, and low mortality rates. However, the study highlights the necessity for confirmation through phase 3 randomized trials to delineate the role of PIPAC, evaluate bidirectional therapy, and optimize both treatment strategies and patient selection criteria.

Therefore, we present, to our knowledge, the first systematic review with a meta-analysis of PIPAC for GCPM. Specifically, our goal consisted in assessing the role of PIPAC in the treatment of PM in GC, thus addressing its feasibility, safety, and efficacy. 

Firstly, there is uniformity across studies regarding the PIPAC technique and the intraperitoneal chemotherapy regimens. Along with detailing an almost identical procedural approach, all studies in this review predominantly employ the same chemotherapy during PIPAC. Almost all studies administered a combination of cisplatin 7.5 mg/m^2^ and doxorubicin 1.5 mg/m^2^, except Kurtz et al., which used a higher dose of cisplatin 15 mg/m^2^ and doxorubicin 1.5 mg/m^2^ and Sgarbura et al., which administered oxaliplatin at a dose of 92 mg/m^2^. Concerning Ellebæk et al., a subset of patients received cisplatin 7.5 mg/m^2^ and doxorubicin 1.5 mg/m^2^ in combination with electrostatic precipitation. It has been shown that drug distribution after PIPAC might not be ideal. Numerous studies conducted ex vivo and in animal models have demonstrated an elevation in drug gradient from the upper to the lower regions of the target volume, coupled with a decline from the axis of the aerosolizing device towards the periphery. The incorporation of electrostatic precipitation, referred to as ePIPAC, mitigated the impact of gravity and enhanced the uniformity of drug distribution. Following ePIPAC, there was an observed rise in tissue drug concentration towards the electrode responsible for generating the electrostatic gradient [11]. Thus, ePIPAC might consist in an attractive path for further development of PIPAC, to improve the homogeneity of spatial distribution and depth of tissue penetration.

In fact, all 18 included studies approach PIPAC as a palliative treatment, in which the presence of PM is an inclusion criterion. Nonetheless, Graversen et al. published in 2023 the PIPAC-OPC4 study, which aimed to evaluate the feasibility and safety of prophylactic PIPAC administration after D2 gastrectomy in patients with gastric cancer at a high risk of recurrence [71]. In fact, they intended to determine whether PIPAC cisplatin (10.5 mg/m^2^ body surface in 150 mL saline) and doxorubicin (2.1 mg/m^2^ body surface in 50 mL saline) would be able to eliminate free intraperitoneal cancer cells after D2 gastrectomy, and thereby improve prognosis for patients. They concluded that Laparoscopic D2 gastrectomy in combination with PIPAC is feasible and safe.

Regarding our study, feasibility was assessed and confirmed, and a considerable number of studies reported a non-access rate of 0%. In fact, all 18 included studies considered synchronous and metachronous PM, hence a considerable number of patients included had already received primary tumor surgical resection or extended surgery, such as cytoreductive surgery and HIPEC for peritoneal metastasis. Concerning the length of hospitalization, the meta-analysis demonstrated patients who underwent three or more PIPAC procedures had a shorter length of hospital stay, when compared to patients who received one or two PIPAC procedures. Considering hospitalization did not increased with repeated treatment procedures, one could assume that prolonged hospitalization was not necessary, aiding in demonstrating that the procedure proved itself feasible. A meta-analysis was attempted regarding PIPAC operative time; however, moderated heterogeneity was observed between studies, and no significant differences were observed.

When assessing the safety of PIPAC, 12 of the 18 included studies entailed data regarding the occurrence of adverse events post-surgery. In fact, when stated, most studies reported no occurrence of deaths related to the procedure. Moreover, the recent literature has documented cases of 30-day post-operative mortality, unrelated to the procedure but linked to the clinical deterioration of highly fragile patients. These early mortality cases, stemming from compromised general conditions, underscore the importance of refined patient selection. In this context, experts reached a consensus on regarding a life expectancy of less than 3 months, recent episodes of bowel occlusion, inability to orally feed, reliance on parenteral nutrition, and an Eastern Cooperative Oncology Group Performance Statues (ECOG PS) score greater than 3 as contraindications to PIPAC. Additionally, a meta-analysis was conducted to assess the overall occurrence of adverse events in patients who underwent three or more PIPAC sessions, comparing them to patients who completed one or two PIPAC sessions. However, the analysis revealed moderate heterogeneity, and no significant differences were observed.

Indeed, the evaluation of treatment response, and consequently, the efficacy of the PIPAC procedure, is a focal point in numerous prospective and retrospective studies. This assessment stands as one of the primary endpoints in all phase II trials.

In fact, PRGS was the most prevalent outcome regarding the efficacy assessment. We conducted a meta-analysis concerning 299 patients of three different studies. When comparing patients who underwent three or more PIPAC procedures with patients who underwent one or two PIPAC procedures, our meta-analysis revealed a significantly higher prevalence of complete or partial histopathological response in patients who underwent multiple procedures, when compared with patients who completed one or two PIPAC procedures.

However, several concerns pertaining to pathological response, as highlighted in the recent literature, warrant attention. Primarily, there is notable variability in the methods employed for assessing pathological response. In our study, emphasis was solely placed on the PRGS score. Additionally, certain studies differentiate in reporting this outcome within the Per Protocol (PP) or Intention to Treat (ITT) populations, potentially introducing bias. Furthermore, a significant portion of studies exclusively assess pathological response in patients who have undergone at least two PIPAC sessions, leading to histologic response rates that may not fully represent the entire study population. Lastly, considerable heterogeneity exists in the regimens administered to patients undergoing PIPAC procedures. While some receive concomitant systemic chemotherapy, offering potential added benefits, others strictly adhere to the PIPAC protocol alone. Unfortunately, these differences could not be analyzed separately, potentially introducing bias to some extent.

The Peritoneal Cancer Index (PCI) is extensively utilized for the pre-operative evaluation of the extent of peritoneal disease. In recent years, it has also been explored as an alternative means of assessing chemotherapy response. 

The visual assessment of PCI was consistently reported at the initial PIPAC session in all examined studies, but its documentation as a response outcome throughout repeated cycles was infrequent. A meta-analysis was conducted in 174 patients with respect to a cut-off of PCI 12 or higher, but no significant differences were observed (RR = 1.02; 95%CI (0.80; 1.30); *p* = 0.873), despite the absence of detected heterogeneity (I^2^ = 0%; *p* = 0.53).

Nevertheless, the visual assessment of peritoneal disease extent is subject to operator-dependent variability, and in patients undergoing chemotherapy, it might not accurately reflect the vitality of actual tumor nodules. Due to these limitations, we believe that relying solely on visual PCI has limited utility in the current context of multimodal treatment response assessment. Further investigation in future studies is necessary to better understand its potential role.

Concerning the survival analysis of GC patients who underwent PIPAC, 12 studies report median OS, with Nadiradze et al., reporting a median OS of 15.4 months.

When documented, a substantial proportion of the included studies present survival percentages up to 18 months, with certain studies extending the reported survival data to 24 months.

In addition, we performed a meta-analysis on the mean OS, combining data from two distinct studies that included a total of 188 patients. Notably, we observed significant differences, revealing that patients undergoing repeated procedures had a higher mean OS compared to those who underwent one or two PIPAC sessions (MD = 6.0; 95%CI (4.2; 7.8); *p* < 0.001), and no heterogeneity was detected (I^2^ = 0%; *p* = 0.52). These conclusions support the evidence reported in the recent literature, which indicates an increase in survival with the amount of PIPAC delivered. In a multicentered cohort study involving 586 patients with GCPM, Alyami et al. reported a median survival of 15.4 months from diagnosis. For patients who underwent more than three PIPAC sessions, the median survival extended to 20.1 months [72]. In addition, Balmer et al. documented a significantly extended median OS among patients who underwent more than three PIPAC sessions compared to those in the PIPAC < 3 patient group (16 versus 7.2 months, *p* < 0.001) within a cohort of 183 patients with PM originating from various primary tumor origins [73]. Nonetheless, the meta-analysis performed on the mean OS presents a significant limitation that warrants attention. The two included studies in this meta-analysis pertain to populations with a wide PCI range: 16.5 ± 8.7 (interquartile range of 2–36) [44] and 17.2 ± 7.4 (interquartile range of 1–39) [38]. PIPAC is recommended for patients with a PCI > 12 in cases of the intestinal histological type of gastric cancer. For the diffuse type (signet ring cell carcinoma) the PCI cutoff is generally lower (PCI > 7). This likely explains the broad PCI range observed, as both studies included in the meta-analysis include patients with both intestinal and diffuse types of carcinomas. This substantial heterogeneity among the study populations may impact the survival analysis.

Ideally, a stratified survival analysis should be conducted, categorizing the population based on tumor type (diffuse vs. intestinal). However, this stratified analysis is not feasible due to insufficient available data.

In fact, in our systematic review, we included, when stated, the percentage of patients who underwent three or more PIPAC procedures. We report that the rate of three or more PIPAC range from 0 to 100% in all included studies, achieving a 100% completion in a study consisting of only one GC patient, included in a cohort of patients concerning several primary tumor origins—moreover, some authors have defined per-protocol treatment as the patient’s completion of three or more cycles of PIPAC. Conversely, a few studies solely evaluate a single PIPAC procedure, disregarding the consideration of repeated procedures in their design. This underscores the substantial variability present across studies.

This study emphasized the emerging evidence regarding the concurrent utilization of PIPAC alongside courses of systemic chemotherapy. Notably, apart from one study, all included studies in our systematic review consider PIPAC as a combined treatment approach. While initial assessments primarily evaluated PIPAC as a monotherapy, the current trend in expert centers involves administering PIPAC concurrently with systemic treatment. While the feasibility of this combination appears evident, its safety needs clarification through specifically designed phase II studies [74,75].

Furthermore, an increasing volume of literature is exploring the use of PIPAC as a neoadjuvant treatment preceding curative surgery. Two of the included studies have specifically investigated this aspect.

In our systematic review, we documented a total of 18 patients from the included studies who became eligible for CRS-HIPEC following PIPAC procedures. This underscores that, despite PIPAC being primarily employed as a palliative treatment, in selected cases, it could play a pivotal role in the conversion to secondary CRS-HIPEC, thus possibly enabling a future curative intent.

Given the potential limitations of a single PIPAC course, meticulous patient selection becomes imperative. Investigating prognostic factors influencing the administration of multiple PIPAC cycles could refine our current selection criteria. A hypothesis worth exploring is that initiating PIPAC earlier in the progression of peritoneal carcinomatosis might allow for multiple cycles, potentially improving overall survival. Current research also delves into the role of PIPAC in a first-line setting, before the onset of resistance to systemic chemotherapy, and, as suggested by Graversen et al., even before the manifestation of macroscopic peritoneal dissemination [71].

In conclusion, it is essential to acknowledge that while our meta-analysis indicates a positive and beneficial role of PIPAC in treating patients with GCPM, this study is not without limitations. Certain aspects require more detailed examination to guide future research in this area.

Firstly, it is essential to highlight that all the studies incorporated into our analysis comprised either retrospective or prospective cohorts, apart from a retrospective case–control study and three phase II clinical trials. Despite an exhaustive search, we determined that there has not been a single randomized controlled trial published to date. Consequently, the absence of randomization in the primary studies included in this meta-analysis may introduce confounding linked to indication bias. This bias might result in patients with a more favorable prognosis being inclined to undergo multiple PIPAC cycles compared to other patients with greater comorbidities, who are more likely to discontinue treatment.

Secondly, our aim was to assess the PIPAC procedure exclusively in patients with gastric cancer who developed PM. Throughout our thorough investigation, we identified only a total of 11 studies that specifically concentrated on this tumor origin. Despite this limitation, we made diligent efforts to gather the most comprehensive data possible.

Moreover, whenever possible, we collected data on patients with gastric cancer from studies that included a variety of tumor origins. Nevertheless, this approach may present a limitation as it could introduce substantial heterogeneity across studies. Notably, sample sizes varied widely, ranging from 1 patient retrieved from a study with mixed cohorts to 144 patients in a specific cohort focusing on GCPM. Consequently, during our meta-analysis, we observed moderate heterogeneity in several of the outcomes examined.

## 5. Conclusions

This systematic review and meta-analysis were able to suggest the feasibility, safety, and effectiveness of PIPAC as a palliative approach in carefully selected patients with GCPM. The findings demonstrate that, with repeated cycles, patients may achieve partial or complete histopathological response, leading to an extended mean overall survival without exacerbating hospitalization. In certain cases, PIPAC might serve as a bridge, enabling the conversion to CRS-HIPEC, thereby improving the prognosis for PM.

Numerous ongoing phase II and phase III randomized trials are actively exploring the efficacy of PIPAC across various contexts, including first-line, adjuvant, or neoadjuvant applications. To gain a comprehensive understanding of PIPAC’s impact on the natural progression of GC, additional randomized controlled trials, with a specific emphasis on GCPM, are essential. Ensuring the robustness of these studies requires the implementation of a more rigorous patient selection process.

## Figures and Tables

**Table 1:** Included studies in the systematic review and their characteristics. N = participants; % = percentage; SD = standard deviation; GC = gastric cancer; OC = ovarian cancer; CRC = colorectal cancer; MT = mesothelioma; PPC = primary peritoneal cancer; HBPC = hepatobiliary pancreatic cancer; APC = appendiceal cancer; PMP = pseudomyxoma peritonei; CUP = cancer unknown primary; YSC = yolk sac cancer; PC = prostate cancer; SCT = systemic chemotherapy; CRS = cytoreductive surgery; RAM = ramucirumab; TGAS = total gastrectomy; ^1^ = due to an anaphylactic reaction to platin, one patient received doxorubicin monotherapy; ^2^ = mean number of PIPAC (SD); ^3^ = median ± SD. (xx; xx)^4^ = number of patients submitted to HIPEC and/or CRS after PIPAC procedures. N/A = not applicable.

Author, Year	Country	Type of Study	Data Collection Period	Sample Size (N)	PIPAC Sessions (N)	PIPAC Chemotherapy	Female (%)	Age, Mean ± SD	PCI, Mean ± SD	Follow-Up (Months), Mean ± SD	Primary Tumor	PIPAC Regimen
Alyami et al., 2021 [34]	Saudi Arabia	Retrospective cohort	N/A	42	163	Cisplatin (7.5 mg/m^2^) + Doxorubicin (1.5 mg/m^2^)	52.4	52.3 ± 10.0	18.5 ± 8.7	N/A	GC	(PIPAC + SCT) + CRS − HIPEC (6) ^4^
Tidadini et al., 2021 [47]	France	Retrospective case–control	07/2016–09/2020	17	42	Cisplatin (7.5 mg/m^2^) + Doxorubicin (1.5 mg/m^2^)	41.2	63.0 ± 3.4	17.0 ± 2.2	16.1 ± 12.0	GC	(PIPAC + SCT/SCT only) + CRS − HIPEC (2; 1) ^4^
Di Giorgio et al., 2020 [35]	Italy	Retrospective cohort	09/2017–09/2019	28	46	Cisplatin (7.5 mg/m^2^) + Doxorubicin (1.5 mg/m^2^)	57.1	(50.0 ± 14.1) ^3^	18.8 ± 7.2	N/A	GC	(PIPAC + SCT) + CRS − HIPEC (1) ^4^
Gockel et al., 2018 [44]	Germany	Prospective cohort	11/2015–06/2018	24	46	Cisplatin (7.5 mg/m^2^) + Doxorubicin (1.5 mg/m^2^)	37.5	58.3 ± 8.0	16.5 ± 8.7	8.4 ± 4.8	GC	(PIPAC + SCT/PIPAC only) + CRS − HIPEC (2) ^4^
Nadiradze et al., 2015 [36]	Germany	Retrospective cohort	N/A	24	60	Cisplatin (7.5 mg/m^2^) + Doxorubicin (1.5 mg/m^2^)	50	56.0 ± 13.0	16.0 ± 10.0	11.3 ± 5.5	GC	PIPAC + SCT/PIPAC only
Khomyakov et al., 2016 [48]	Russia	Phase II clinical Trial	08/2013–06/2016	31	56	Cisplatin (7.5 mg/m^2^) + Doxorubicin (1.5 mg/m^2^)	71	49.8 ± 11.6	18.0 ± 6.8	N/A	GC	PIPAC + SCT
Feldbrügge et al., 2021 [37]	Germany	Retrospective cohort	03/2017–05/2020	50	90	Cisplatin (7.5 mg/m^2^) + Doxorubicin (1.5 mg/m^2^)	45	55.8 ± 11.0	19.5 ± 8.5	N/A	GC	(PIPAC + SCT/PIPAC + SCT + RAM) + CRS − HIPEC (5) ^4^
Struller et al., 2019 [49]	Germany	Phase II clinical trial	11/2013–04/2016	25	43	Cisplatin (7.5 mg/m^2^) + Doxorubicin (1.5 mg/m^2^)	60	55.1 ± 13.0	15.3 ± 10.6	N/A	GC	PIPAC only
Sindayigaya et al., 2021 [38]	Germany	Retrospective cohort	N/A	144	296	Cisplatin (7.5 mg/m^2^) + Doxorubicin (1.5 mg/m^2^)	52	56.0 ± 14.7	17.2 ± 7.4	18.0 ± 11.4	GC	PIPAC + SCT/PIPAC only
Ellebæk et al., 2020 [33]	Denmark	Prospective cohort	03/2015–10/2018	20	52 (11 ePIPAC + 41 PIPAC)	Cisplatin (7.5 mg/m^2^) + Doxorubicin (1.5 mg/m^2^) + electrostatic precipitation	65	54.5 ± 7.4	15.5 ± 9.9	12.7 ± 6.2	GC	(e)PIPAC + SCT/(e)PIPAC only
Horvath et al., 2022 [39]	Germany	Retrospective cohort	04/2016–09/2021	44	93	Cisplatin (7.5 mg/m^2^) + Doxorubicin (1.5 mg/m^2^) ^1^	50	50.8 ± 14.7	23.5 ± 8.7	N/A	GC	N/A
Račkauskas et al., 2021 [40]	Lithuania	Retrospective cohort	2015–2020	9	N/A	Cisplatin (7.5 mg/m^2^) + Doxorubicin (1.5 mg/m^2^)	N/A	N/A	N/A	N/A	GC OC	N/A
Sgarbura et al., 2019 [41]	France	Retrospective cohort	01/2015–12/2017	15	(2.6 (0.8)) ^2^	Oxaliplatin 92 mg/m^2^	N/A	N/A	19 ± 5.2	N/A	GC OC CRC	N/A
Somashekhar et al., 2019 [45]	India	Prospective cohort	06/2017–12/2017	1	3	Cisplatin + Doxorubicin	N/A	N/A	11.0	N/A	GC OC CRC MT PPC	N/A
Tidadini et al., 2022 [42]	France	Retrospective cohort	07/2016–09/2020	18	N/A	Cisplatin (7.5 mg/m^2^) + Doxorubicin (1.5 mg/m^2^)	N/A	N/A	N/A	N/A	GC CRC Other	(PIPAC+ SCT) + TGAST + CRS + HIPEC (2) ^4^
Kurtz et al., 2018 [43]	Germany	Retrospective cohort	N/A	26	N/A	Cisplatin (15 mg/m^2^) + Doxorubicin (1.5 mg/m^2^)	N/A	N/A	N/A	N/A	GC HBPC OC APC PMP CUP MT YSC PC	PIPAC + SCT/PIPAC only
De Simone et al., 2020 [50]	Italy	Phase II clinical trial	10/2015–12/2018	7	N/A	Cisplatin (7.5 mg/m^2^) + Doxorubicin (1.5 mg/m^2^)	N/A	N/A	N/A	N/A	GC CRC MT OC PMP PPC	PIPAC+ SCT/PIPAC only
Katdare et al., 2018 [46]	India	Prospective cohort	05/2017–08/2017	2	2	Cisplatin (7.5 mg/m^2^) + Doxorubicin (1.5 mg/m^2^)	0.0	80.0	5.0	N/A	GC OC APC CRC	N/A

**Table 2:** Feasibility outcomes. % = percentage; SD = standard deviation; N/A = not applicable. * = median.

Author, Year	Non-Access (%)	≥3 PIPAC (%)	Operative Time (Minutes), Mean ± SD	Hospital Stay (Days), Mean ± SD
Alyami et al., 2021 [34]	N/A	71.4	N/A	16.0 ± 12.4
Tidadini et al., 2021 [47]	N/A	N/A	N/A	3.3 ± 1.4
Di Giorgio et al., 2020 [35]	4.0	15.2	124.0 * ± 40.3	2.0 * ± 0.5
Gockel et al., 2018 [44]	9.6	45.8	109.5 ± 18.5	4.8 ± 1.8
Nadiradze et al., 2015 [36]	6.7	41.7	91.0 ± 34.0	N/A
Khomyakov et al., 2016 [48]	0.0	25.0	N/A	3.0
Feldbrügge et al., 2021 [37]	N/A	26.0	75.8 ± 17.6	11.3 ± 9.1
Struller et al., 2019 [49]	2.0	24.0	N/A	N/A
Sindayigaya et al., 2021 [38]	13.9	29.7	62.3 ± 14.1	5.7 ± 2.8
Ellebæk et al., 2020 [33]			ePIPAC—71.5 ± 13.9;	
	0.0	50.0	PIPAC—98.0 ± 22.6	N/A
Horvath et al., 2022 [39]	0.0	27.3	113.8 ± 38.1	N/A
Račkauskas et al., 2021 [40]	0.0	N/A	N/A	N/A
Somashekhar et al., 2019 [45]	0.0	100.0	N/A	2.0
Katdare et al., 2018 [46]	0.0	0.0	109.0	2.0

**Table 3:** Safety outcomes. % = percentage; N = participants; N/A = not applicable.

Author, Year	CTCAE 3–4 (%)	CTCAE 5 (%)	Clavien–Dindo 3a, 3b or 4 (%)	Sample Size (N)
Alyami et al., 2021 [34]	9.2	4.8	N/A	42
Tidadini et al., 2021 [47]	N/A	N/A	11.8	17
Di Giorgio et al., 2020 [35]	4.0	0.0	N/A	28
Gockel et al., 2018 [44]	N/A	N/A	0.0	24
Nadiradze et al., 2015 [36]	29.2	8.3	N/A	24
Khomyakov et al., 2016 [48]	3.2	0.0	N/A	31
Feldbrügge et al., 2021 [37]	N/A	N/A	6.0	50
Struller et al., 2019 [49]	12.0	0.0	N/A	25
Sindayigaya et al., 2021 [38]	2.4	0.6	N/A	144
Ellebæk et al., 2020 [33]	5.0	0.0	N/A	20
Somashekhar et al., 2019 [45]	0.0	0.0	N/A	1
Katdare et al., 2018 [46]	0.0	0.0	N/A	2

**Table 4:** Survival analysis outcomes. OS = overall survival; % = percentage; CI = confidence interval; N = participants; N/A = not applicable.

Authors, Year	Median OS, Months (95% CI)	%OS at 6 Months	%OS at 12 Months	%OS at 18 Months	%OS at 24 Months	Sample Size (N)
Alyami et al., 2021 [34]	N/A	97.8	73.5	55.7	39.4	42
Tidadini et al., 2021 [47]	12.8 (7.2–34.3)	94.1 (65.0–99.2)	94.1 (65.0–99.2)	51.8 (26.2–72.4)	38.8 (16.3–61.1)	17
Di Giorgio et al., 2020 [35]	12.3 (11.7–17.4)	N/A	N/A	N/A	N/A	28
Gockel et al., 2018 [44]	7.0 (2.2–20.8)	60.0	39.0	19.3	N/A	24
Nadiradze et al., 2015 [36]	15.4	69.1	61.1	N/A	N/A	24
Khomyakov et al., 2016 [48]	13.0	85.9	56.2	42.6	0.0	31
Struller et al., 2019 [49]	6.7 (2.5–12.0)	54.4	24.0	N/A	N/A	25
Sindayigaya et al., 2021 [38]	11.0 (0.0–61.0)	77.2	39.2	24.6	14.4	142
Ellebæk et al., 2020 [33]	11.5	100.0	44.5	17.8	8.7	20
Horvath et al., 2022 [39]	6.0 (1.4–21.2)	N/A	N/A	N/A	N/A	44
Račkauskas et al., 2021 [40]	8.0 (4.0–16.0)	55.6	44.9	0.0	0.0	9
Sgarbura et al., 2019 [41]	N/A	81.5	67.9	0.0	0.0	15
Tidadini et al., 2022 [42]	6.0 (2.9–15.5)	89.0	49.1	36.9	36.9	18
Kurtz et al., 2018 [43]	6.8	56.7	0.0	0.0	0.0	26
De Simone et al., 2020 [50]	N/A	85.8	35.8	35.8	0.0	7

## Data Availability

The data for this study are based on the published literature and are available upon request from the corresponding author.

## Supplementary Materials

The following supporting information can be downloaded at: https://www.mdpi.com/article/10.3390/jcm13113320/s1, Table S1. Search terms and queries; Table S2. Description of the answers in the NIH (National Institutes of Health) quality assessment criteria for observational studies. Y = yes; N = no; NR = not reported; Table S3. Description of the answers in the NIH (National Institutes of Health quality assessment criteria for case-control studies. Y = yes; N = no; NR = not reported; Table S4. Description of the answers in the NIH (National Institutes of Health quality assessment criteria for before-after (pre-post) studies with no control group. Y = yes; N = no; NR = not reported; NA = not applicable.
