# Supplementary material for: Pressurized Intraperitoneal Aerosol Chemotherapy (PIPAC) in the Treatment of Gastric Cancer: Feasibility, Efficacy and Safety—A Systematic Review and Meta-Analysis
Source: J Clin Med. 2024 Jun 4;13(11):3320. doi: 10.3390/jcm13113320 (PMC11173180; doi:10.3390/jcm13113320)
Supplement: Supplementary file 1 [file jcm-13-03320-s001.zip › jcm-3001271-supplementary.pdf]

Table S1: Search terms and queries.

Databases	Queries
MEDLINE/ Pubmed	(((Pressurized Intraperitoneal Aerosol Chemotherapy[MeSH Terms]) OR (PIPAC[Title/Abstract]) OR (Intraperitoneal[Title/Abstract]) OR ("Intraperitoneal Aerosol Chemotherapy"[Title/Abstract])) AND (("gastric cancer"[Title/Abstract]) OR ("stomach cancer"[Title/Abstract]) OR ("gastric adenocarcinoma"[Title/Abstract]) OR ("stomach adenocarcinoma"[Title/Abstract]))) AND ((peritoneal neoplasms[MeSH Terms]) OR (carcinosis[Title/Abstract]) OR (carcinomatosis[Title/Abstract]) OR ("peritoneal metastasis"[Title/Abstract]) OR ("peritoneal metastases"[Title/Abstract]) OR (peritoneal diseases[MeSH Terms]))
Web of Science	TS=("Pressurized Intraperitoneal Aerosol Chemotherapy" OR PIPAC OR intraperitoneal OR "Intraperitoneal Aerosol Chemotherapy") AND TS=("gastric cancer" OR "stomach cancer OR "gastric adenocarcinoma" OR "stomach adenocarcinoma" OR "gastric neoplasm" OR "stomach neoplasm") AND TS=("peritoneal neoplasms" OR "peritoneal cancer" OR carcinosis OR carcinomatosis OR "peritoneal metastasis" OR "peritoneal metastases")
SCOPUS	(TITLE-ABS-KEY(Pressurized Intraperitoneal Aerosol Chemotherapy) OR TITLE-ABS-KEY(PIPAC) OR TITLE-ABS-KEY(Intraperitoneal) OR TITLE-ABS-KEY(Intraperitoneal Aerosol Chemotherapy)) AND (TITLE-ABS-KEY(gastric cancer) OR TITLE-ABS-KEY(stomach cancer) OR TITLE-ABS-KEY(gastric adenocarcinoma) OR TITLE-ABS-KEY(stomach adenocarcinoma)) AND (TITLE-ABS-KEY(peritoneal neoplasms) OR TITLE-ABS-KEY(carcinosis) OR TITLE-ABS-KEY(carcinomatosis) OR TITLE-ABS-KEY(peritoneal metastasis) OR TITLE-ABS-KEY(peritoneal metastases) OR TITLE-ABS-KEY(peritoneal diseases)) AND (LIMIT-TO (PUBYEAR,2023) OR LIMIT-TO (PUBYEAR,2022) OR LIMIT-TO (PUBYEAR,2021) OR LIMIT-TO (PUBYEAR,2020) OR LIMIT-TO (PUBYEAR,2019) OR LIMIT-TO (PUBYEAR,2018) OR LIMIT-TO (PUBYEAR,2017) OR LIMIT-TO (PUBYEAR,2016) OR LIMIT-TO (PUBYEAR,2015) OR LIMIT-TO (PUBYEAR,2014) OR LIMIT-TO (PUBYEAR,2013) OR LIMIT-TO (PUBYEAR,2012) OR LIMIT-TO (PUBYEAR,2011)) AND (LIMIT-TO (LANGUAGE,"English") OR LIMIT-TO (LANGUAGE,"Portuguese"))

Table S2: Description of the answers in the NIH (National Institutes of Health) quality assessment criteria for observational studies. Y=yes; N=no; NR=not reported.

Author, Year	1	2	3	4	5	6	7	8	9	10	11	12	13	14	Y/N (NR)
Alyami *et al.*, 2021	Y	Y	Y	Y	N	Y	Y	N	Y	Y	Y	NR	Y	N	10/3 (1)
Di Giorgio *et al.*, 2020	Y	Y	Y	Y	N	Y	Y	Y	Y	Y	Y	NR	Y	N	11/2 (1)
Gockel *et al.*, 2018	Y	Y	Y	Y	N	Y	Y	Y	Y	Y	Y	NR	Y	N	11/2 (1)
Nadiradze *et al.*, 2015	Y	Y	Y	Y	N	Y	Y	N	Y	Y	Y	NR	Y	N	10/3 (1)
Feldbrügge *et al.*, 2021	Y	Y	Y	Y	N	Y	Y	N	Y	Y	Y	NR	Y	N	10/3 (1)
Sindayigaya *et al.*, 2021	N	Y	Y	Y	N	Y	Y	Y	Y	Y	Y	NR	Y	Y	11/2 (1)
Ellebæk *et al.*, 2020	Y	Y	Y	Y	N	Y	Y	Y	Y	Y	Y	NR	Y	N	11/2 (1)
Horvath *et al.*, 2022	Y	Y	Y	Y	N	Y	Y	Y	Y	Y	Y	NR	Y	N	11/2 (1)
Račkauskas *et al.*, 2021	Y	Y	Y	Y	N	Y	Y	Y	Y	Y	Y	NR	Y	N	11/2 (1)
Sgarbura *et al.*, 2019	Y	N	Y	N	N	Y	Y	Y	Y	Y	Y	NR	Y	Y	10/3 (1)
Somashekhar *et al.*, 2019	Y	Y	Y	Y	N	Y	Y	N	Y	Y	Y	NR	Y	N	10/3 (1)
Tidadini *et al.*, 2022	Y	Y	Y	Y	N	Y	Y	Y	Y	Y	Y	NR	Y	N	11/2 (1)
Kurtz *et al.*, 2018	Y	Y	Y	Y	N	Y	Y	Y	Y	Y	Y	NR	Y	Y	12/1 (1)
Katdare *et al.*, 2018	Y	Y	Y	Y	N	Y	N	N	Y	Y	Y	NR	Y	N	9/4 (1)

1 - Was the research question or objective in this paper clearly stated?
2 – Was the study population clearly specified and defined?
3 – Was the participation rate of eligible persons at least 50%?
4 – Were all the subjects selected or recruited from similar populations? Were inclusion and exclusion criteria for being in the study prespecified and applied uniformly to all participants?
5 - Was a sample size justification, power description, or variance and effect estimates provided?
6 - For the analyses in this paper, were the exposure(s) of interest measured prior to the outcome(s) being measured?
7 - Was the timeframe sufficient so that one could reasonably expect to see an association between exposure and outcome if it existed?
8 - For exposures that can vary in amount or level, did the study examine different levels of the exposure as related to the outcome?
9 - Were the exposure measures (independent variables) clearly defined, valid, reliable, and implemented consistently across all study participants?
10 - Was the exposure(s) assessed more than once over time?
11 - Were the outcome measures (dependent variables) clearly defined, valid, reliable, and implemented consistently across all study participants?
12 - Were the outcome assessors blinded to the exposure status of participants?
13 - Was loss to follow-up after baseline 20% or less?
14 - Were key potential confounding variables measured and adjusted statistically for their impact on the relationship between exposure(s) and outcome(s)?

Table S3: Description of the answers in the NIH (National Institutes of Health) quality assessment criteria for case–control studies. Y=yes; N=no; NR=not reported.

Author, Year	1	2	3	4	5	6	7	8	9	10	11	12	Y/N (NR)
Tidadini *et al.*, 2021	Y	Y	N	Y	Y	Y	NR	Y	Y	Y	NR	Y	9/1 (2)

1 - Was the research question or objective in this paper clearly stated and appropriate?
2 – Was the study population clearly specified and defined?
3 – Did the authors include a sample size justification?
4 – Were controls selected or recruited from the same or similar population that gave rise to the cases (including the same timeframe)?
5 - Were the definitions, inclusion and exclusion criteria, algorithms or processes used to identify or select cases and controls valid, reliable, and implemented consistently across all study participants?
6 - Were the cases clearly defined and differentiated from controls?
7 - If less than 100 percent of eligible cases and/or controls were selected for the study, were the cases and/or controls randomly selected from those eligible?
8 - Was there use of concurrent controls?
9 - Were the investigators able to confirm that the exposure/risk occurred prior to the development of the condition or event that defined a participant as a case?
10 - Were the measures of exposure/risk clearly defined, valid, reliable, and implemented consistently (including the same time period) across all study participants?
11 - Were the assessors of exposure/risk blinded to the case or control status of participants?
12 - Were key potential confounding variables measured and adjusted statistically in the analyses? If matching was used, did the investigators account for matching during study analysis?

Table S4: Description of the answers in the NIH (National Institutes of Health) quality assessment criteria for before–after (pre–post) studies with no control group. Y=yes; N=no; NR=not reported; NA=not applicable.

Author, Year	1	2	3	4	5	6	7	8	9	10	11	12	Y/N (NR)
Khomyakov *et al.*, 2016	Y	Y	Y	Y	N	Y	Y	NR	Y	N	Y	NA	8/2 (1)
Struller *et al.*, 2019	Y	Y	Y	Y	Y	Y	Y	NR	Y	Y	Y	NA	10/0 (1)
De Simone *et al.*, 2020	Y	Y	Y	N	Y	Y	Y	Y	Y	Y	Y	NA	10/1 (0)

1 - Was the study question or objective clearly stated?
2 -Were eligibility/selection criteria for the study population prespecified and clearly described?
3 -Were the participants in the study representative of those who would be eligible for the test/service/intervention in the general or clinical population of interest?
4 -Were all eligible participants that met the prespecified entry criteria enrolled?
5 -Was the sample size sufficiently large to provide confidence in the findings?
6 -Was the test/service/intervention clearly described and delivered consistently across the study population?
7 - Were the outcome measures prespecified, clearly defined, valid, reliable, and assessed consistently across all study participants?

8 - Were the people assessing the outcomes blinded to the participants' exposures/interventions?
9 - Was the loss to follow-up after baseline 20% or less? Were those lost to follow-up accounted for in the analysis?

10 - Did the statistical methods examine changes in outcome measures from before to after the intervention? Were statistical tests done that provided p values for the pre-to-post changes?
11 -Were outcome measures of interest taken multiple times before the intervention and multiple times after the intervention (i.e., did they use an interrupted time-series design?
12 - If the intervention was conducted at a group level (e.g., a whole hospital, a community, etc.) did the statistical analysis take into account the use of individual-level data to determine effects at the group level?
